# Draft Genome Sequence of “*Rathayibacter tanaceti*” Strain VKM Ac-2596 Isolated from *Tanacetum vulgare* Infested by a Foliar Nematode

**DOI:** 10.1128/genomeA.00512-16

**Published:** 2016-06-16

**Authors:** Oleg V. Vasilenko, Irina P. Starodumova, Sergey V. Tarlachkov, Lubov V. Dorofeeva, Alexander N. Avtukh, Lyudmila I. Evtushenko

**Affiliations:** a All-Russian Collection of Microorganisms (VKM), GK Skryabin Institute of Biochemistry and Physiology of Microorganisms, Pushchino, Russia; b Pushchino State Institute of Natural Sciences, Pushchino, Russia; c Branch of Shemyakin and Ovchinnikov Institute of Bioorganic Chemistry, Pushchino, Russia

## Abstract

The draft genome of “*Rathayibacter tanaceti*” VKM Ac-2596 is 3.17 Mb in size with an average G+C content of 70.7% and comprises at least two nonidentical copies of ribosomal small subunit (SSU-rRNA) genes. The semiconductor sequencing platform Ion Torrent was used.

## GENOME ANNOUNCEMENT

The genus *Rathayibacter* comprises six validly described species, including plant pathogens (*R. rathayi, R. iranicus, R. tritici* and *R. toxicus*), which are transmitted to their host plants (cereals and grasses, *Poaceae*) by gall-forming nematodes of the genus *Anguina* (*Anguinidae*) (1, 2). Type strains of the two other *Rathayibacter* spp. have been isolated from *Festuca rubra* (*Poaceae*) infected by *Anguina graminis* and from *Carex* sp. (*Cyperaceae*) without any symptoms of nematode infestation (3). Unlike the above species, strain VKM Ac-2596 originates from a plant of the family *Asteraceae*, *Tanacetum vulgare*, infested by the foliar nematode *Aphelenchoides fragariae* (*Aphelenchoididae*). The strain exhibited 99.6% 16S rRNA gene sequence similarity to *R. rathayi*, *R. iranicus*, and *R. tritici*, while the concordant results from matrix-assisted laser desorption ionization–time of flight mass spectra clustering and multilocus phylogenetic analysis (*gyr*B, *rec*A, *rpo*B, and *ppk*) suggest that it represents a novel species, provisionally named “*Rathayibacter tanaceti*” (unpublished data). The availability of the whole-genome sequence of this bacterium will facilitate insight into the genomic basis for the species delineation in plant-associated bacteria and the exploration of molecular mechanisms related to plant pathogenicity of *Rathayibacter*-nematode complexes.

The sequencing of strain VKM Ac-2596 was performed with the semiconductor genome analyzer Ion Torrent PGM (Thermo Fisher Scientific Inc., USA) using a 400-bp sequencing kit and a 318 v2 chip. A total of 373,614 raw reads were assembled *de novo* into 331 contigs (17.0-fold peak coverage) using Newbler version 3.0 (454 Life Sciences Corporation, USA). The genome size is 3,168,884 bp with an average G+C content of 70.7%. The *N*_50_ contig is 20,949 bp, and the largest contig is 69,183 bp. The open reading frames and rRNA sequences were predicted and annotated using Prokka version 1.11 (4) with the Barrnap version 0.5 plugin (http://www.vicbioinformatics.com/software.barrnap.shtml). A total of 3,209 protein-encoding genes (1,038 of which have no similarity to sequences in current databases), 52 tRNAs, 1 tmRNA, and 3 rRNAs were predicted. Apparently there was one copy of the SSU-rRNA gene, but the Sanger partial sequence revealed nucleotide ambiguity (A or G) at position 23 (accession no. KU891049). The mapping of PGM primary reads on the draft genome assembly (Bowtie2 version 2.2.3 [5]) showed that the average coverage was 35.8 for the SSU locus, whereas it was 18.7 for the total genome assembly. Relevant primary reads contained either A (36 reads) or G (14 reads) but neither T nor C. The data are indicative of the presence of at least two nonidentical 16S rRNA gene copies in the genome of the target strain. To our knowledge, only a single copy or two identical copies of the 16S rRNA gene have been revealed for *Rathayibacter* spp. so far. This follows from our survey of the rRNA operon copy number database (*rrn*DB) (6) and simple BLASTn search within the eight *Rathayibacter* genomes deposited at DDBJ/EMBL/GenBank (accession nos. GCA_000425325.1, GCA_000875675.1, GCA_001465855.1, GCA_000986985.1, GCA_001423045.1, GCA_001423885.1, GCA_001423005.1, and GCA_001423055.1). This paper reports the first case of the 16S rRNA gene ambiguity in *Rathayibacter* spp*.*

### Nucleotide sequence accession numbers.

This whole-genome shotgun project has been deposited at DDBJ/EMBL/GenBank under the accession number LIIN00000000. The version described in this paper is the first version, LIIN01000000.
